# Calicivirus Infection in Cats

**DOI:** 10.3390/v14050937

**Published:** 2022-04-29

**Authors:** Regina Hofmann-Lehmann, Margaret J. Hosie, Katrin Hartmann, Herman Egberink, Uwe Truyen, Séverine Tasker, Sándor Belák, Corine Boucraut-Baralon, Tadeusz Frymus, Albert Lloret, Fulvio Marsilio, Maria Grazia Pennisi, Diane D. Addie, Hans Lutz, Etienne Thiry, Alan D. Radford, Karin Möstl

**Affiliations:** 1 Clinical Laboratory, Department of Clinical Diagnostics and Services, Vetsuisse Faculty, University of Zurich, 8057 Zurich, Switzerland; hans.lutz@uzh.ch; 2 MRC—University of Glasgow Centre for Virus Research, Glasgow G61 1QH, UK; margaret.hosie@glasgow.ac.uk; 3 Clinic of Small Animal Medicine, Centre for Clinical Veterinary Medicine, Ludwig Maximilian University of Munich, 80539 Munich, Germany; hartmann@lmu.de; 4 Department of Biomolecular Health Sciences, Faculty of Veterinary Medicine, University of Utrecht, 3584 CL Utrecht, The Netherlands; h.f.egberink@uu.nl; 5 Institute of Animal Hygiene and Veterinary Public Health, University of Leipzig, 04103 Leipzig, Germany; truyen@vetmed.uni-leipzig.de; 6 Bristol Veterinary School, University of Bristol, Bristol BS40 5DU, UK; s.tasker@bristol.ac.uk; 7 Linnaeus Veterinary Limited, Shirley, Solihull B90 4BN, UK; 8 Department of Biomedical Sciences and Veterinary Public Health (BVF), Swedish University of Agricultural Sciences (SLU), P.O. Box 7036, 750 07 Uppsala, Sweden; sandor.belak@slu.se; 9 Scanelis Laboratory, 31770 Colomiers, France; corine.boucraut@scanelis.com; 10 Department of Small Animal Diseases with Clinic, Institute of Veterinary Medicine, Warsaw University of Life Sciences—SGGW, 02-787 Warsaw, Poland; tadeusz_frymus@sggw.edu.pl; 11 Fundació Hospital Clínic Veterinari, Universitat Autònoma de Barcelona, Bellaterra, 08193 Barcelona, Spain; albert.lloret@uab.es; 12 Faculty of Veterinary Medicine, Università degli Studi di Teramo, 64100 Teramo, Italy; fmarsilio@unite.it; 13 Dipartimento di Scienze Veterinarie, Università di Messina, 98168 Messina, Italy; mariagrazia.pennisi@unime.it; 14 Veterinary Diagnostic Services, School of Veterinary Medicine, College of Medical, Veterinary and Life Sciences, University of Glasgow, Glasgow G61 1QH, UK; draddie@catvirus.com; 15 Veterinary Virology and Animal Viral Diseases, Department of Infectious and Parasitic Diseases, Fundamental and Applied Research for Animals & Health Research Centre, Faculty of Veterinary Medicine, Liège University, B-4000 Liège, Belgium; etienne.thiry@uliege.be; 16 Institute of Infection, Veterinary and Ecological Sciences, University of Liverpool, Leahurst Campus, Chester High Road, Neston CH64 7TE, UK; a.d.radford@liverpool.ac.uk; 17 Institute of Virology, Department for Pathobiology, University of Veterinary Medicine, 1210 Vienna, Austria; karinmoestl@gmail.com

**Keywords:** FCV, feline, multicat environment, genetic variability, virulent systemic, diagnosis, vaccination, vaccine strains, tenacity, treatment

## Abstract

Feline calicivirus (FCV) is a common pathogen in domestic cats that is highly contagious, resistant to many disinfectants and demonstrates a high genetic variability. FCV infection can lead to serious or even fatal diseases. In this review, the European Advisory Board on Cat Diseases (ABCD), a scientifically independent board of experts in feline medicine from 11 European countries, presents the current knowledge of FCV infection and fills gaps with expert opinions. FCV infections are particularly problematic in multicat environments. FCV-infected cats often show painful erosions in the mouth and mild upper respiratory disease and, particularly in kittens, even fatal pneumonia. However, infection can be associated with chronic gingivostomatitis. Rarely, highly virulent FCV variants can induce severe systemic disease with epizootic spread and high mortality. FCV can best be detected by reverse-transcriptase PCR. However, a negative result does not rule out FCV infection and healthy cats can test positive. All cats should be vaccinated against FCV (core vaccine); however, vaccination protects cats from disease but not from infection. Considering the high variability of FCV, changing to different vaccine strain(s) may be of benefit if disease occurs in fully vaccinated cats. Infection-induced immunity is not life-long and does not protect against all strains; therefore, vaccination of cats that have recovered from caliciviral disease is recommended.

## 1. Introduction

Feline calicivirus (FCV) is a highly contagious pathogen with a widespread distribution in the feline population [1]. It can be found in asymptomatic carriers [2]; at the same time, FCV infection can lead to several distinct clinical problems. Frequently and typically, it is found in association with oral ulcers, particularly of the tongue and palate, as well as with mild upper respiratory signs [3]. In kittens, and also rarely in adult cats, FCV infection can lead to severe pneumonia [4]. A limping syndrome with shifting lameness of different limbs has been documented during acute FCV infection as well as after FCV immunisation with live-attenuated virus vaccines [5,6]. Moreover, FCV has been associated with feline chronic gingivostomatitis [7], and finally, virulent systemic FCV (VS-FCV) infection has been described with often lethal outcome for the affected cat, or for several cats within a multicat environment due to epizootic spread of the disease [8]. Thus, FCV is a virus that presents many facets of clinical problems. This can partially be due to the high genetic variability [9], although no association between particular genetic characteristics or mutations of FCV and clinical presentation has been proven to date [10]. FCV infection is particularly widespread in multicat environments [11] and a problem when associated with the previously mentioned multiple clinical problems that FCV infection can provoke. Although the virus has been known and investigated for more than 60 years [12], there are still many unsolved puzzles, such as a lack of vaccines that induce sterilising immunity or that protect reliably from VS-FCV-induced disease. The current review gives an update on the current state of knowledge on FCV.

## 2. Virus

FCV belongs to the *Caliciviridae* family, genus *Vesivirus*. Caliciviruses include important pathogens of humans (such as the norovirus, one of the commonest causes of infectious gastroenteritis in people) and of other animal species, including the European brown hare syndrome virus and rabbit haemorrhagic disease virus [13].

**Figure 1:**
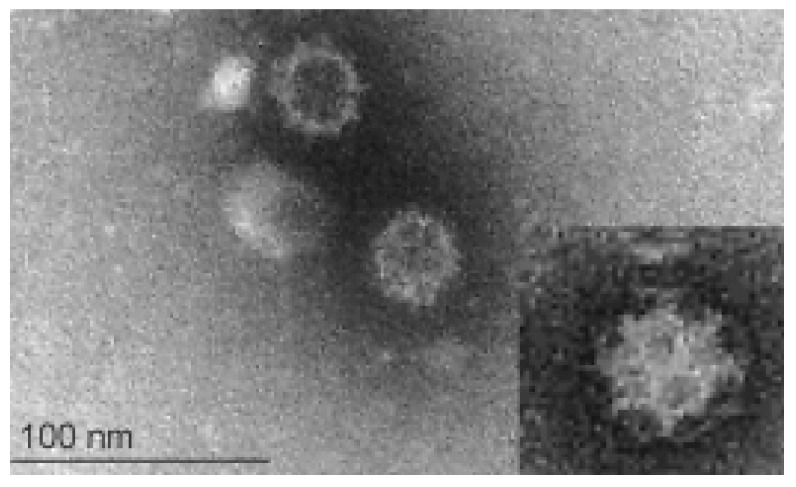
At high magnification, the eponymic goblets (calices) are visible on the virion surface. © Marian C. Horzinek.

Calicivirus particles are hexagonal or star shaped and show cup-shaped depressions in electron microscopic preparations; their name is derived from the Greek *calyx* meaning cup or goblet (Figure 1).

**Figure 2:**
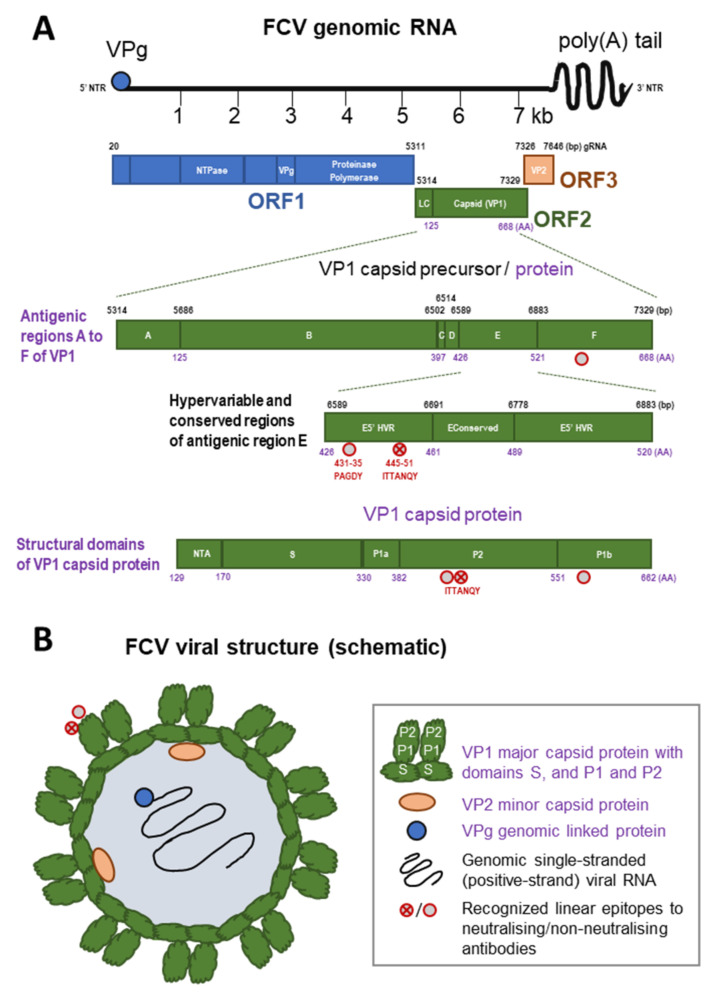
(**A**) Genomic organisation of FCV genomic RNA with open reading frames (ORF) 1 to 3, antigenic regions (A to F) of the VP1 capsid precursor protein, hypervariable and conserved regions of antigenic region E and scheme of the structural domains of the capsid protein VP1 (N-terminal arm [NTA], shell [S], and protruding domain P with subdomains P1 and P2). (**B**) Schematic representation of the viral structure of FCV. Linear non-neutralising epitopes were recognised in the capsid region F and in a conserved area of E5′HVR; moreover, linear epitopes to neutralising antibodies were recognised in E5′HVR [16,17,18] (indicated by red circles; see figure legend). VPg = virus protein, genome linked. NTR = non-translated region. NTPas = nucleoside-triphosphatase. LC = capsid leader. HVR = hypervariable region. Not drawn to scale. Combined and adapted from [17,19,20,21].

The virus has a small (approximately 7.5 kb) single-stranded RNA genome of positive (messenger) polarity, which allows it to evolve quickly. It is a non-enveloped virus and the lack of a lipid envelope contributes to its persistence in the environment and explains why the virus does not fuse with cellular phospholipid membranes of host cells but uses alternative paths to enter them. The genomic RNA (gRNA) of FCV is non-segmented and contains three functional open reading frames (ORF) (Figure 2A). ORF1 encodes a polyprotein that is post-translationally cleaved into non-structural proteins (protease, polymerase, etc.) [14], while ORF2 encodes a polyprotein that is subsequently processed to release a small protein, the leader of the capsid, and the major capsid protein (VP1), and ORF3 encodes the minor capsid protein (VP2) [15]. ORF2 is divided into six regions, A to F, of which region E contains a 5′ hypervariable region (HVR), a central conserved region, and a 3′ HVR (Figure 2A).

The FCV gRNA is enclosed by 90 dimers of the capsid protein VP1 (Figure 2B), the immunodominant protein which is targeted by the host’s immune response [17,18,22,23]. Each capsid protein, VP1, monomer consists of the structural domains N-terminal arm (NTA), shell (S), and protruding domain (P) consisting of the subdomains P1 and P2; at the same time, the six antigenic regions A to F of VP1 have been described (Figure 2A). Region E (P2) is responsible for binding of the cellular receptor (feline junctional adhesion molecule A, fJAM-A) as well as immune recognition by the host [17,24]. The cellular receptor of FCV, fJAM-A, is located at tight junctions of endothelial and epithelial cells and regulates the integrity and permeability of these cell layers. Disruption of the tight junctions leads to oral and cutaneous ulcerations. In addition, fJAM-A is also present on feline platelets and peripheral leukocytes. After binding of VP1 to fJAM-A, the virus undergoes confirmational changes that are necessary for infection of the cell [24]. VP2 is critical for releasing the viral genome through the endosomal membrane into the cytoplasm of the host cell by formation of a portal-like assembly during the first stages of infection, and it is essential for production of infectious virions [25]. Interactions of VP1 and VP2 are established using the residues Y451, D452, A454 and D455 of VP1 [25]. The latter are in close proximity to epitopes characterised in the E5′ HVR of subdomain P2 of VP1. A linear epitope that was recognised by neutralising antibodies is located at amino acids 445 to 451 (ITTANQY) and a non-neutralising epitope is located at amino acids 431 to 435 (PAGDY). In addition, a linear epitope recognised by non-neutralising antibodies was found in region F of VP1 [17] (Figure 2). The E5′ and E3′ HVR are considered targets for immune evasion during persistent FCV infection, and point mutations were found in neutralisation escape mutants within or close to the neutralisation epitope ITTANQY [22]. In contrast, and although also within the hypervariable region, the epitope PAGDY was found to be rather well conserved, and will react with most FCV isolates, and antibodies to this region were broadly cross-reacting and might have the potential for further exploration for FCV diagnostics [17].

Antigenic differences between different FCV isolates can create some difficulties when trying to maximise vaccine cross protection [26]. Nonetheless, most strains of FCV are sufficiently closely related antigenically to induce some degree of cross protection and this has been utilised in developing vaccines. Genetically, most FCVs belong to a single diverse genogroup [10,27,28]; however, a second genogroup has been described in East Asia [19,29,30,31]. In investigation of the spatial and temporal distribution of FCV strains, a very high genetic and antigenic FCV strain complexity has been described, with many different FCV strains circulating within a cat population, and no individual field strain dominating it [28,32]. Based on this observation, it was suggested that FCV evolution is not associated with selective competition among different strains in the cat population [28]. No association has been demonstrated between FCV capsid gene sequences or antigenic characteristics and different disease manifestations [10,26,33,34,35]. 

## 3. Epidemiology

There are no known reservoirs or alternative hosts for FCV, apart from wild felids. Humans are not susceptible to infection. As well as the existence of a specific canine calicivirus, FCV-like viruses have been isolated occasionally from dogs [36,37,38,39]. The role of these FCV-like viruses in the epidemiology of FCV in both dogs and cats is uncertain [40,41], but is probably not significant.

FCV is shed by cats with acute disease predominantly via oral and nasal secretions but it is also detected in the blood, urine and faeces of infected cats. On recovery, many cats continue shedding, most of them for at least 30 days post-infection, and a few for several years up to life-long [42]. A small proportion of cats were found to be non-shedders, although continuously exposed to FCV over long periods; these cats were thought to be resistant to infection, probably dependent on immune-mediated mechanisms or genetic host factors [43].

FCV infection is widespread in the general cat population. The prevalence is broadly proportional to the number of cats in a household. It is lowest in healthy household cats kept in small groups of fewer than four cats (2.5%), and higher in groups of healthy cats of four or more cats (32%) [2,44]. The prevalence within individual colonies and shelters is variable, ranging from low to high (50–90%) values [11,41,43,45,46]. A study in stray cat colonies in Italy found that 85% of the cats were antibody positive for FCV [47]. No association was found in an Australian study between socioeconomic status of the owners and the prevalence of FCV infection, which contrasted with findings for feline immunodeficiency virus (FIV) infection [48]. 

High FCV prevalence within a colony is associated with high FCV strain diversity, related to a small number of persistently infected cats and reinfection of other members of the cat population with either a variant of the same FCV strain or with a different strain [9,46]. Each cat colony or group is usually infected with distinct viruses that evolve from distinct ancestors [46,49]. At the same time, individual cats can be infected with more than one FCV strain [9,50]. Introduction of new strains into a cat colony and infection of individual cats with more than one strain can lead to recombination events, increasing genetic variability; the latter might be associated with the selection of antigenic variants in a cat that escape the collective immune response, thereby enabling endemic infection in a cat population [9,51]. In shelter environments with a high turnover of cats and, in turn, with frequent introductions of new FCV strains, particularly high genetic variability of FCV has been demonstrated [33]. In comparison, a low level of viral diversity of FCV was found within stable multicat households [33]. The spread of FCV within cat shelters might be reduced by good hygiene and biosecurity measures [52]. Infection generally occurs through direct contact with secretions from acutely infected and carrier cats [53]. However, the virus survives in the environment and remains infectious for up to one month on dry surfaces at room temperature, and even longer in colder conditions [54,55,56]. The virus also persists longer in damp compared to dry environments, and aerosol spread has been detected in facilities with FCV-shedding cats [34]. Indirect transmission can occur, especially within the close confines of a cattery, where secretions can contaminate cages, feeding and cleaning tools, or personnel. Moreover, indirect transmission via fomites or people must also be expected in facilities, as has been described for VS-FCV variants [8,57,58]. In one study, a primary role of caregivers’ hands in the spread of a VS-FCV outbreak was reported; although washing and disinfecting procedures had been in place, it was assumed that one student infected up to ten cats during physical examination or oral medication of cats [59]. The virus can also remain infectious in flea faeces for up to eight days, and kittens were experimentally infected with FCV by contact with infected fleas or their faeces [60].

## 4. Pathogenesis

Cats can be infected with FCV via the nasal, oral or conjunctival routes. The oropharynx is the primary site of replication. Transient viraemia occurs three to four days after infection—at which time, the virus is detected in many other tissues. The virus induces necrosis of epithelial cells: vesicles, typically on the tongue, develop into ulcers; in the affected regions, the mucosa is infiltrated with neutrophils. Healing usually takes place over a period of two to three weeks [61], but can last significantly longer in individual cases.

**Figure 3:**
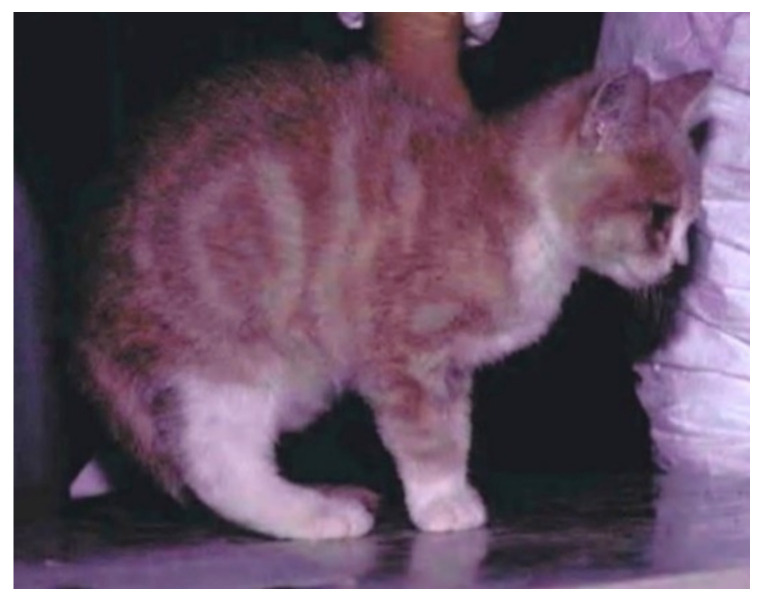
Calicivirus infection limping syndrome. ©Uwe Truyen.

FCV less commonly affects other tissues, such as the lungs or joints, leading to pneumonia (focal alveolitis, progressing to areas of acute exudative pneumonia and then to proliferative, interstitial pneumonia) and lameness, the so-called limping syndrome (Figure 3). Co-infections of feline herpesvirus (FHV) and FCV have been described in kittens with pneumonia; FHV infection leads to airway damage, which might facilitate secondary infection with FCV due to reduced mucociliary clearance and impaired immune defences [62]. Acute synovitis with thickening of synovial membranes and increase in the amount of synovial fluid have been noted in cats with limping syndrome [5]. The pathogenesis of the limping syndrome is not clear, although immune complexes are thought to play a role [63], and FCV can be isolated from affected joints [5]. Limping syndrome can also occur after FCV vaccination with some modified live virus vaccines [6].

The pathogenesis and clinical presentation of systemic disease caused by VS-FCV differ considerably from the typical picture described above. These VS-FCV strains cause widespread vasculitis, multiorgan involvement, and death in up to two-thirds of infected cats [3,8,57,64]. The pathogenesis of VS-FCV infection is unknown and might include viral evolution and/or immune-mediated components as well as environmental and management factors [65]. The virulent strains display a different cell tropism and grow more rapidly in cell culture compared to less virulent isolates [66,67]. The faster in vitro growth was associated with the capsid protein gene of the virulent FCV isolate [24]. The authors of the latter study suggested that conserved surface residues on the P2 subdomain of the major capsid protein VP1 (Figure 2) are important for the interaction of FCV with the cellular receptor, fJAM-A, and subsequent conformational changes that likely influence the in vitro infection kinetics and the virulence of different FCV isolates in vivo [24] (see also Section 2).

Healthy cats can be FCV carriers [2,43,68]; in these cats, the virus can be localised mainly in the retropharyngeal area, close to the tonsils. However, in an experimental setting, tonsillectomy did not eliminate the carrier state, suggesting that virus is also located outside of the tonsils [69]. It is believed that evolution of the variable capsid protein allows FCV to escape the host immune response and to persist in carrier cats [9,70,71,72] although some structural restrictions might prevent substantial antigenic evolution of the FCV capsid [73]. Indeed, it has been shown that two residues of the capsid protein VP1 (P2; amino acids 459 and 462) are essential for binding of FCV to its cellular receptor, fJAM-A, while seven residues are suggested to be necessary for subsequent events after binding [24,25]. Along these lines, the epitope PAGDY in subdomain P2 (amino acids 431 to 435) of the capsid protein VP1 (Figure 2), was found to be highly conserved and mutation of amino acids 431 and 433 lead to non-viable mutants [17,24]. Other mechanisms by which FCV might evade the host’s immune response include the degradation of mRNAs (RNase effect) by the viral proteinase-polymerase protein, which leads to inhibition of gene expression; and with that, it contributes to host gene shutoff [74], and the blocking of the type I interferon-induced antiviral innate immunity by the non-structural p30 protein of certain FCV strains [75].

## 5. Immunity

### 5.1. Passive Immunity by Maternally Derived Antibodies

Maternally derived antibodies (MDA) are important for protection during the first weeks of life and can interfere with vaccination. There are sparse data on the extent and longevity for FCV MDA in cats. In general, their levels are higher and persist for longer than for FHV. In an experimental study, the average half-life of FCV MDA was determined to be 15 days and their persistence as 10–14 weeks [76]. However, in a field study, 20% of kittens at only six weeks of age had no detectable antibodies against a widely used FCV vaccine strain [77].

### 5.2. Active Immune Response

Virus-neutralising antibodies appear by approximately seven days post-infection [78]. One linear epitope recognised by neutralising antibodies is located in the 5′ HVR of the antigenic region E of VP1 (subdomain P2) spanning amino acids 445 to 451 (ITTANQY, Figure 2). Neutralisation escape was found to be related to mutations in or close to this epitope, at amino acids 441, 448, 449 and 455 [22]. Other neutralising, as well as linear, B-cell epitopes were so far all mapped to the major capsid protein VP1 [16,17,79]. 

In general, FCV-neutralising antibody titres are higher than for FHV infection and their levels correlate well with protection against homologous challenge [80]. Although protection against heterologous strains is not always present, prior infection with one strain can still significantly reduce the acute clinical signs upon exposure to a heterologous strain, and in some cases oral shedding can be reduced [34,80,81,82]. In general, the level of heterologous protection will depend on the virus strains involved. The more antigenically diverse the strains are in relation to the vaccine strain(s), the lower the expected cross protection might be (see also Section 9.1). Cats can also be protected in the absence of detectable virus-neutralising antibodies [81,83,84], suggesting a role for other immune mechanisms, and indeed, cellular responses have been demonstrated in vaccinated cats [82,85]. Additionally, FCV-specific IgG and IgA antibodies have been found in the saliva during the course of infection [81], although their significance in protection is unknown.

## 6. Clinical Signs

FCV infection can cause acute oral and upper respiratory signs but also has been associated with chronic gingivostomatitis, which is considered an immune-mediated condition. Moreover, “foot and paw” and VS-FCV disease have been described.

### 6.1. Acute Oral and Upper Respiratory Tract Disease

**Figure 4:**
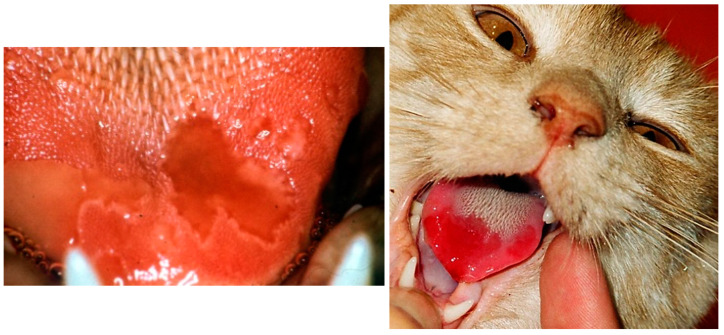
Ulcerative lesions on the tongue of cats with FCV infection. Left, ©Marian C. Horzinek; right, ©Tadeusz Frymus.

Clinical findings can differ, depending on the FCV strain, the age of the affected cats and husbandry factors. While in some cases infection is subclinical, in many others, there is a typical syndrome of lingual ulcers (Figure 4) and a relatively mild acute respiratory disease. More severe signs can resemble the respiratory disease caused by FHV; however, frequently occurring co-infections with FHV, *Chlamydia felis,* or *Mycoplasma felis* might be responsible for some of the respiratory signs rather than FCV itself [2].

**Figure 5:**
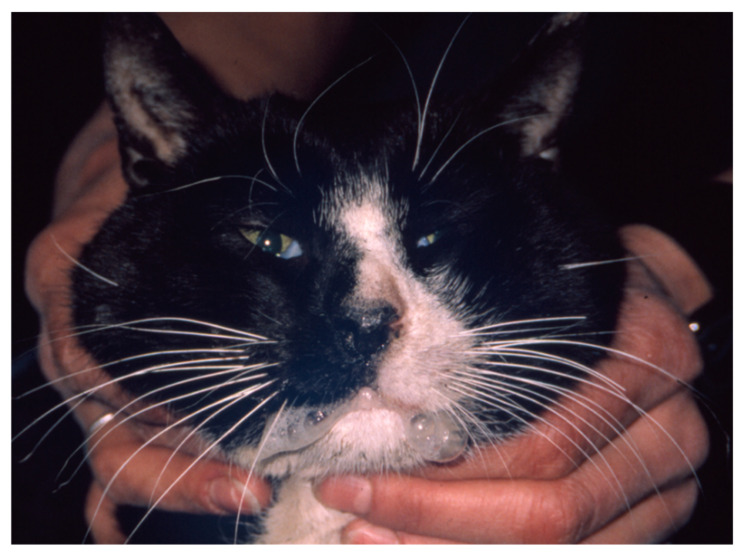
Hypersalivation in a cat with FCV infection. Tongue ulceration can be very painful, leading to impaired food and water uptake and hypersalivation. ©Tadeusz Frymus.

Acute oral and upper respiratory disease signs are mainly seen in kittens. The incubation period is two to ten days [3]. Oral ulcerations, sneezing, and serous nasal discharge are the main signs [61]. Fever is also commonly observed. Anorexia, sometimes accompanied by hypersalivation (Figure 5) due to oral ulcers (located mainly on the tongue), is often more prominent than signs of rhinitis [2,86]. Signs usually resolve after a few days with only symptomatic treatment [87,88].

In some severe cases, pneumonia, manifested by dyspnoea, coughing, fever, and depression, can occur, particularly in young kittens. Moreover, cases of erosive conjunctivitis have been reported in FCV-infected cats [89]. In rare cases, cats with corneal ulcers had high viral FCV RNA loads in the absence of other pathogens, i.e., FHV, *Mycoplasma felis* and *Chlamydia felis*, that could have been related to the lesions (C.B.-B. personal communication). However, it is unclear what the role of FCV is in these cases.

### 6.2. Feline Chronic Gingivostomatitis (FCGS)

**Figure 6:**
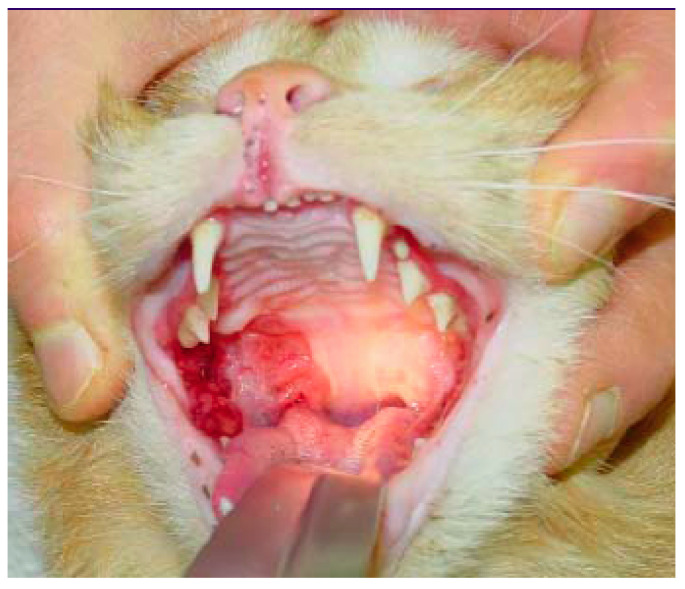
Feline chronic gingivostomatitis. ©University Animal Hospital Zurich, Felicitas Boretti.

An excellent review on periodontal disease and FCGS (Figure 6) has been published [90], and this cautions that severe gingivitis in a cat infected with FCV does not automatically provide for a diagnosis of FCGS. The inflammation in FCGS, by definition, extends beyond the mucogingival junction to encompass the alveolar mucosa and other soft tissues. If the inflammation is confined to gingival tissues, by definition, a diagnosis of FCGS cannot be made. The aetiology of FCGS remains uncertain and several infectious agents can be associated with FCGS. In a prospective study, FCV was more commonly detected by reverse-transcriptase PCR (RT-PCR) in 52 cats with FCGS (54%) than in 50 healthy age-matched control cats (24%) [91]. Similar results were found in a study investigating 42 oral biopsies of cats with FCGS (40% FCV RT-PCR positive versus none of the 19 controls) [92]. Additional studies also confirmed these observations in a larger population of cats in Spain (in 260 cats with FCGS, 59% FCV positive versus in 98 controls, 15% FCV positive) [93] and in cats in Japan [94]. Likewise, using unbiased metagenomic and transcriptomic analyses of 23 cats with FCGS and 19 control cats, the only microbe strongly associated with FCGS was FCV [7]. However, the FCV RNA loads in FCV-positive cats with FCGS were not associated with disease severity [95]. In contrast to the majority of published studies, no FCV antigen was found in FCGS lesions of 26 cats examined by immunohistochemistry [96]. 

It has been suggested that FCGS is an immune-mediated reaction to FCV (and potentially other oral antigens). The disease has not been reproduced experimentally using two different FCV isolates from cats with FCGS for oronasal infection (Knowles et al., 1991). Moreover, FCV isolates from cats with different acute or chronic respiratory disease signs or from cats with chronic oral lesions induced an acute disease with fever, apathy, oral ulcers, gingivitis, rhinitis, and/or conjunctivitis when experimentally inoculated in kittens, indicating that chronicity is probably more related to the host immune response than to any particular biotype of FCV [26]. FCV does not appear to play a role in feline odontoclastic resorptive lesions (FORLs) [97].

### 6.3. Limping Syndrome

An acute transient shifting lameness (Figure 3) with fever can be associated with FCV infection [5,98,99] and can also occur after FCV vaccination [6]. Experimental oronasal infection with the FCV vaccine strain 255 lead to mostly mild lameness in a small number of cats (3/10) [99]. Some cases developed following vaccination with modified live FCV vaccines using different strains. It is not always the vaccine strain that is accountable for the limping, since in some cases FCV field strain that were different to vaccine strains were also detected and vaccination was incidental [6]. In natural infection, limping syndrome can occur a few days or weeks after the acute oral or respiratory signs. The lameness can be severe and can shift quickly from one limb to another. Full recovery usually occurs within 24–48 h without treatment, but NSAIDs can be useful to reduce inflammation and pain.

### 6.4. Paw and Mouth Disease

Paw and mouth disease has been reported in Australia [100] prior to the description of VS-FCV infections in the US and Europe. It initially very much resembled VS-FCV disease but high mortality and epizootic spread were not observed [100,101]. Characteristic clinical signs include cutaneous oedema and ulcerative lesions on the skin of paws and on the head, in and around the mouth, and in the perianal region, as well as fever, depression and anorexia [100,101]. Oedema is located mainly on the head and limbs. Usually, mortality is not high, but fatal cases have also been described [102,103]. In contrast to VS-FCV, “paw and mouth” disease is seen in single or very few cats, and no epizootic spread of the disease is observed [100,101,102,103,104]. It is unclear whether this was a distinct syndrome or whether these were also mild outbreaks of VS-FCV disease.

### 6.5. Virulent Systemic Feline Calicivirus Infection

Outbreaks of highly virulent and often lethal FCV infection in domestic cats have been described in the United States [3,8,57,67,105], China [106,107] and in several European countries: France [58,59], Germany [108], Italy [109], Switzerland (R.H.-L. personal communication), the UK [64], and Australia [35]. A single outbreak has also been described in exotic captive felids in the United States [110]. The disease was initially named “hemorrhagic-like fever” [8] but subsequently “highly virulent feline calicivirus disease” was suggested, since haemorrhages were rarely observed [57,105]. The causative virus strains are most commonly referred to as “virulent systemic feline calicivirus” (VS-FCV).

Phylogenetic analyses of VS-FCV and FCV from cats with other FCV-associated clinical signs or asymptomatic cats did not reveal clustering according to the different clinical presentations; thus, there was no clear association between disease severity and virus genetic background and no mutations could be identified that clearly distinguished different clinical presentations [35]. Therefore, RT-PCR or sequencing of FCV cannot be used to confirm or rule out virulent systemic disease per se; the clinical signs and the epidemic situation should also be considered before making such a diagnosis.

The incubation period in natural cases of VS-FCV infection in cats exposed in hospitals is usually one to five days; in the home environment it can extend up to 12 days [3]. The disease appears to be more severe in adults than kittens.

In contrast to the common strains, VS-FCV strains cause systemic disease characterised by severe systemic inflammatory response syndrome, disseminated intravascular coagulation (DIC), multiorgan failure, and commonly death. Mortality is high (~30–70%) [57,111]; in one outbreak, 79% (11/14) of the cats had to be euthanised or died with FCV-associated virulent systemic disease [59].

**Figure 7:**
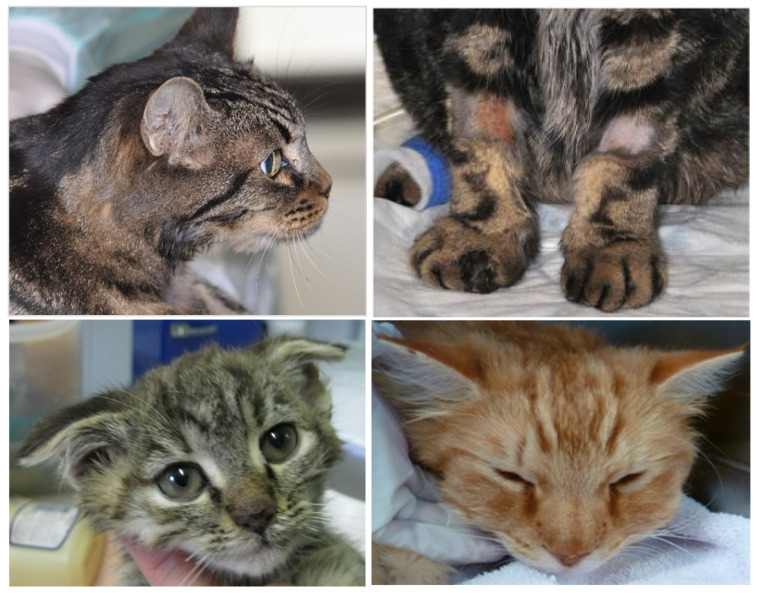
Oedema of the head and limbs in cats with VS-FCV infection. ©University Animal Hospital Zurich, Regina Hofmann-Lehmann.

**Figure 8:**
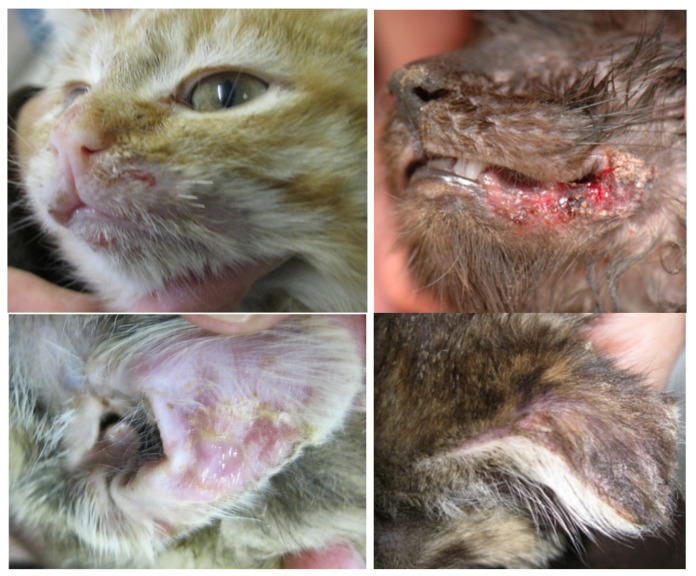
Crusted lesions and ulcers of cats with VS-FCV infection: facial, mucocutaneous (lower lip and commissure) and ears. ©University Animal Hospital Zurich, Regina Hofmann-Lehmann.

**Figure 9:**
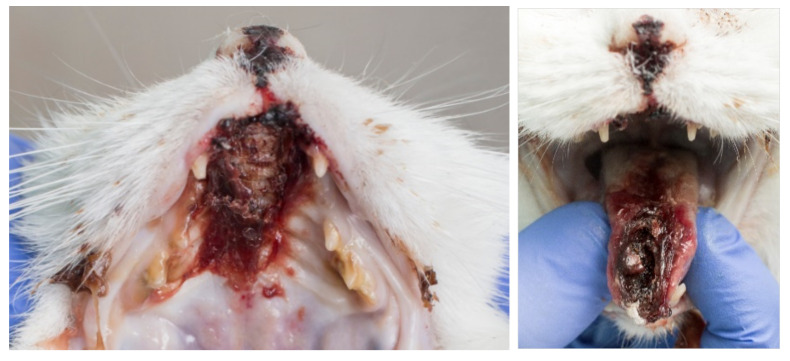
Severe necrosis (philtrum, palate, tongue, lip commissure) in a cat with VS-FCV infection. ©University Animal Hospital Zurich, Regina Hofmann-Lehmann.

**Figure 10:**
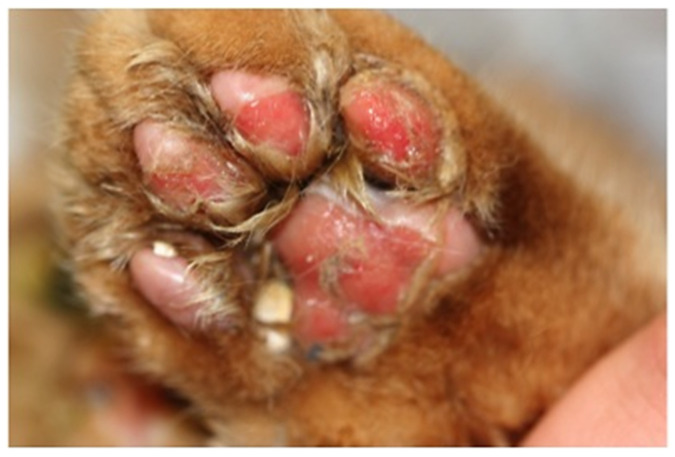
Excoriations of paws in a cat with VS-FCV infection. ©Katrin Hartmann. LMU Munich.

The clinical signs of VS-FCV infection are variable. The initial findings are frequently typical of a severe acute upper respiratory tract disease. Characteristic signs are cutaneous oedema and ulcerative lesions on the skin and paws [3]. Oedema is located mainly on the head and limbs (Figure 7). Crusted lesions, ulcers, and alopecia can be seen on the nose, lips, ears, around the eyes (Figure 8) as well as in the mouth and on the tongue (Figure 9), and on the footpads (Figure 10).

Not all cats with VS-FCV infection show all the typical clinical signs, which makes it difficult to recognize all affected cats.

**Figure 11:**
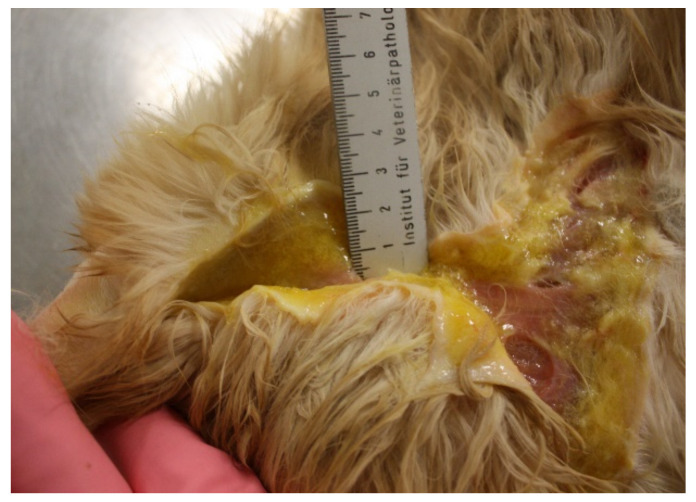
Jaundice and oedema of the subcutaneous tissue at necropsy of a cat with VS-FCV infection (case 11, [102]). ©Institute of Veterinary Pathology, Vetsuisse Faculty Zurich.

Moreover, paw and mouth disease associated with FCV infection is characterised by similar initial clinical signs, including oedema, mouth and skin ulceration, and fever [100,102]. Some of the cats with VS-FCV disease are jaundiced (e.g., due to hepatic necrosis, pancreatitis) (Figure 11); some can show severe respiratory distress (e.g., due to pulmonary oedema). Thromboembolism and coagulopathy caused by disseminated intravascular coagulopathy can be observed, causing petechiae, ecchymoses, epistaxis or bloody faeces [3,64,107]. Typically, VS-FCV outbreaks start in multicat environments, such as animal shelters or veterinary clinics, and are usually characterised by the clinical signs listed above, epizootic spread and a high mortality.

### 6.6. Other Clinical Presentations

FCV has also been implicated in other diseases, such as polyps in the tympanic bulla and cystitis, but evidence for these associations is lacking [112,113]. Moreover, FCV was isolated from faeces of cats with enteritis, lending support to the hypothesis that some FCV strains might acquire enteric tropism and act as enteric pathogen [114,115]. FCV strains from faecal samples collected from Chinese cats were found to belong to genogroups I and II; they were co-circulating with respiratory FCV strains and recombination was suspected, which could have contributed to viral evolution [31]. However, currently, there are insufficient data to determine whether FCV can act as a primary causative agent of gastrointestinal disease, whether it can trigger mechanisms of synergism in co-infections with other enteric pathogens, or whether it is passively shed into the gastrointestinal tract of FCV-infected cats. Alternatively, FCV could simply replicate in the upper respiratory tract, be swallowed and then pass through the alimentary tract and be shed in faeces [31].

## 7. Diagnosis

Asymptomatic cats as well as cats vaccinated with modified live virus vaccines post-vaccination can occasionally shed FCV [116]. Therefore, caution should be taken when interpreting any FCV RT-PCR-positive result because of the poor correlation between the presence of viral RNA and clinical signs [117]. 

### 7.1. Detection of Nucleic Acids

Conventional, nested and real-time RT-PCR assays have been developed to detect FCV RNA in conjunctival and oral swabs, blood, cutaneous scrapings or lung tissue, depending on the clinical form and the outcome of the disease. The diagnostic sensitivity of RT-PCR depends on both the primers used in the PCR and the detected strain, because of the high variability of the viral genome; therefore, molecular assays should be optimised using a large panel of strains to minimise false-negative results [118,119,120]. Despite this, neither of two well-established FCV RT-PCR systems was able to amplify all isolates in a Swiss field study [121]. Multiplex PCR/RT-PCR assays have also been developed that detect FHV and FCV (and sometimes also *Chlamydia felis*) at the same time [122], but such assays might be less sensitive. As well as having the potential to diagnose FCV infection, RT-PCR can provide the means of identifying uniquely the virus strain involved and has proven useful in molecular epidemiology and outbreak investigations. Generally, real-time RT-PCR is recommended over conventional RT-PCR assays for diagnostic purposes due to a higher sensitivity, and—if available—quantitative assays are preferred since they also provide information on the virus load in positive samples. The result of diagnostic FCV RT-PCR must always be interpreted in conjunction with the clinical signs. 

A negative RT-PCR result does not rule out FCV infection. However, in a cat that has typical clinical signs and a positive RT-PCR result, a causal relationship is likely.

Factors, other than the sensitivity and specificity of the assay, that can influence the result of the RT-PCR include the time of sampling, storage, and transport [121]. The detection of FCV by RT-PCR was significantly more likely in swabs from the oropharynx and tongue compared to conjunctival swabs [123]. Since there was no difference for the detection of FHV and *Chlamydia felis* among the different sampling sites, it was recommended to use the oropharynx as the preferred sampling site for detection of all three pathogens, but ideally multiple swabs are collected or multiple sites are collected with the same swab (sequentially starting with the conjunctiva), to maximise the likelihood of detection [123].

A more rapid and robust FCV detection method compared to RT-PCR would be useful, particularly in cases where VS-FCV is suspected, and rapid confirmation is essential for biosafety and epidemiological reasons. A method was developed for the rapid detection of FCV using an enzymatic recombinase amplification (ERA) assay combined with a lateral flow dipstick (LFD) [124]; however, its applicability to a wide range of field isolates and without prior nucleic acid extraction has yet to be evaluated.

### 7.2. Virus Isolation

Virus isolation is a useful method for detecting FCV infection; it indicates the presence of replicating virus and has the advantage of being less influenced by the effect of strain variation than RT-PCR. FCV replicates in cell lines of feline origin; its rapid growth in tissue culture can compromise identification of concurrent FHV infection [125]. Virus can be isolated from nasal, conjunctival, or oropharyngeal swabs [126], but virus isolation can fail due to low numbers of virions in the sample, virus inactivation during transit, or to the presence of antibodies in extracellular fluids that prevent virus replication in vitro [121]. The likelihood of successful virus isolation is maximised if swabs from both conjunctiva and oropharynx are collected [127]. Moreover, combined FCV detection by RT-PCR directly from swabs and after virus isolation further increased sensitivity; neither of the methods alone, RT-PCR nor virus isolation, were able to detect all FCV-positive samples in a Swiss field study [121]. Therefore, a negative FCV test result in a cat suspected to be infected with FCV does not fully exclude FCV infection.

### 7.3. Antibody Detection

FCV antibodies can be detected by virus neutralisation or ELISA [128]. The antibody prevalence is generally very high in cat populations because of natural infection and vaccination [129,130]. Consequently, the presence of specific antibodies is not useful to diagnose infection [126]. Levels of virus-neutralising antibodies can be used to predict whether a cat is protected or not, but must be interpreted with care, as false-negative results can be obtained if virus-neutralising antibodies present in the cat do not cross-react with the laboratory strains used in the test. In addition, titres can appear higher when homologous rather than heterologous virus-antibody pairs are used. When the strain used is not defined, it makes interpretation of the results difficult [77,131,132,133]. 

### 7.4. Diagnosis of VS-FCV Infections

Consistent genetic markers associated with virulence, specifically highly virulent strains involved in VS-FCV, have yet to be identified [32,66,111,134]. One study found that the capsid gene of a virulent FCV isolate was responsible for more rapid in vitro growth kinetics [24] and analysis of the amino acid properties of the E region of VP1 identified seven residue positions (438, 440, 448, 452 and 455 in the E3′ HVR; 465 in the E-conserved region; and 492 in the E5′ HVR; see also Figure 2A) that were believed to be different between different pathotypes [135]. However, this assumption was not confirmed in a subsequent Australian study, where VS-FCV from one outbreak showed the typical mutations described for the VS-FCV pathotype, while VS-FCV from two other outbreaks had either the pattern characteristic for a respiratory pathotype or an intermediate configuration [35]. Thus, further studies are needed to further investigate this hypothesis and currently it should be emphasised that there are no diagnostic assays that specifically detect VS-FCV.

The **diagnosis of VS-FCV** infections relies on clinical signs typical for virulent systemic disease including systemic infection and organ involvement, often the occurrence in multicat environments, high contagiousness of the virus and epizootic spread of the infection, a high mortality rate of the disease, and the isolation of the same FCV strain from the blood, oropharyngeal swabs or cutaneous scrapings from ulcerated lesions of several diseased cats assessed by sequencing hypervariable regions of the capsid gene.

What is mentioned above for FCV is also true for VS-FCV: one negative FCV RT-PCR result does not rule out a VS-FCV infection. **It is important to consider adequate hygienic measures as soon as clinical signs compatible with VS-FCV infection are seen in a cat**, although severe systemic but non-epizootic forms of FCV infections exist including paw and mouth disease with clinical presentations initially similar to VS-FCV-associated disease [102,103,104].

## 8. Disease Management

### 8.1. Treatment of Cats with Acute Upper Respiratory Tract or Oral Disease

Most cats affected by FCV will require little or no specific treatment. However, severely affected cats may benefit from intensive nursing care and supportive therapy. Treatment of dehydration and restoration of electrolyte disturbances by intravenous fluid administration are needed for cats with severe clinical signs. Food intake is extremely important. Many cats with FCV infection do not eat, mainly because of fever and/or pain from ulcers in the oral cavity, sometimes also because of their loss of smell due to nasal congestion. Food can be blended to cause less pain when eating, should be highly palatable, and can be warmed up to increase the smell. Tempting the cat to eat by hand feeding of warm smelly blended foods can be sufficient in some cases. Alternatively, commercial fluid high-energy diets can be used for hand feeding. In addition, appetite stimulants (e.g., mirtazapine; 2 mg/cat PO q 24 h—available as a transdermal preparation—can give q48h if renal or hepatic dysfunction is present) can be given. However, if the cat is not eating for more than three days, placement of a feeding tube and enteral nutrition is indicated. One option is to use naso-oesophageal tube (NOT) feeding; these usually require no sedation for placement but cats with FCV might resent the presence of the tube around the nose; softer silicone tubes are now available that can be used for cats with soreness of the nose and/or pharynx. Sometimes intermittent placing of the NOT for each feed can work better than leaving one in place. In more severe cases and in cats that are expected not to eat within a few days, oesophagostomy tube feeding can be used. These need short general anaesthesia for placement but do additionally allow for medicating and easier feeding via the wider bore tubes which are positioned away from the cat’s face in contrast to NOTs.

Non-steroidal anti-inflammatory drugs can be used to decrease fever and oral pain, but only once dehydration is corrected, and ideally when there is food intake. 

Maropitant is sometimes used in cats with upper respiratory signs, as the blocking of substance P could potentially reduce inflammation and pain, but there is no study proving its efficacy in these conditions.

Inexpensive small nebulisers can be purchased for use in the veterinary clinic (or loaned to owners to use at home) for regular nebulisation therapy with saline (q 4–6 h if possible for 15 min at a time); in the hospital setting, this can be applied close to the cat’s face by placing the cat in an igloo and covering the igloo, nebulising the cat within this enclosed space. Nebulisation can rehydrate the upper respiratory tract, loosen secretions, reduce congestion, and increase comfort. If a nebuliser is not available, an alternative is to provide steam therapy. Owners can do this at home by sitting with their cat in an enclosed bathroom with hot running water/shower. Adequate humidity will be present when the bathroom mirror becomes ‘foggy’.

If there is a nasal discharge, this should be cleaned away several times a day with physiological saline solution, and ointment applied locally around the nostrils. If the nose is severely congested, nasal flushing with saline may help. If there is a mucous nasal discharge, drugs with mucolytic effects (e.g., bromhexine) can be helpful.

At the clinician’s discretion, antibiotics should be given to cats with severe disease (lethargy, anorexia, and pyrexia) and suspected secondary bacterial infection (e.g., mucopurulent nasal discharge). An empirical choice is usually made initially, as bacterial culture of nasal discharge is not useful. It is crucial to use antibiotics with good penetration in the respiratory tract and/or oral cavity. Oral doxycycline (10 mg/kg q24h or 5 mg/kg q 12 h) for seven to ten days is recommended as a first-line treatment in the ISCAID guidelines for acute cases only if fever, lethargy, or anorexia is present concurrently with mucopurulent nasal discharge [136].

### 8.2. Antiviral Therapy of Acute Upper Respiratory Disease

Most antivirals used in veterinary medicine only inhibit replication of DNA viruses or retroviruses, and antivirals for the specific treatment of FCV infections are not available. 

Ribavirin is one of the few antiviral agents able to inhibit FCV replication in vitro; additionally, in vitro, synergistic antiviral effects were described with ribavirin in combination with copper chloride, which has a low cytotoxicity [137]. However, ribavirin appears to be very toxic to cats and side effects have precluded its systemic use [138]. 

In one study, PSSNa polymers (polysodium 4-styrenesulfonates), which are believed to have antiviral activity via electrostatic interactions inhibiting viral adhesion and/or entry into the host cell, had no toxicity in vitro and in vivo, and in vitro had inhibited FCV replication [139]; therefore, the authors of this study believed they had potential for topical treatment against FCV but in vivo efficacy studies are lacking. 

Mefloquine, the antiparasitic agent for humans, demonstrated a marked inhibitory effect on the replication of FCV F9 and seven Australian FCV isolates in vitro, and an additive effect was found in combination with interferon-ω in cell culture [140]. Furthermore, the experimental agent adenosine analogue NITD008 reduced FCV replication in vitro [141]. However, the effectiveness of both mefloquine and NITD008 has not been determined in vivo. Likewise, three natural compounds, icariin, formononetin, and caffeic acid phenethyl ester, inhibited FCV replication in vitro and could have potential as drug candidates, although in vivo data are lacking [142].

In vitro as well as in vivo data are available for some compounds and biologicals. Anti-FCV-specific F(ab’)2 fragments elicited in horses immunised with inactivated FCV showed not only efficient FCV-neutralising activity in vitro but they also had therapeutic (when given subcutaneously one to three days after infection) and prophylactic (when given one day prior to infection) effects in a small trial with experimentally FCV-infected cats [143]; cats that received equine immunoglobulin fragments had lower viral loads and better clinical scores and survival rates than untreated controls. Further studies will be necessary to investigate this approach in the field.

Feline interferon-ω (licensed for the treatment of canine parvovirus, feline leukaemia virus and FIV infections in some European countries) has been shown to inhibit FCV replication in vitro [144,145,146]. There is some suggestion that FCV strains can vary in their sensitivity to interferon [147]. One prospective, randomised, placebo-controlled, double-blinded clinical study investigated whether administration of feline interferon-ω using an owner-friendly protocol (one injection, afterwards topical administration) improved clinical signs in cats with acute feline upper respiratory tract disease caused by FCV and/or FHV and whether this treatment reduced shedding of FCV. Thirty-seven cats showing acute clinical signs caused by FCV and/or FHV were randomly assigned to treatment groups, receiving either placebo or feline interferon-ω (2.5 MU/kg subcutaneously, followed by 0.5 MU topically at 8 h intervals via the conjunctiva, intranasally, and orally for 21 days). All cats received additional symptomatic treatment. All cats showed rapid improvement of clinical signs, but treatment with feline interferon-ω was not more effective in ameliorating clinical signs than placebo [87].

Nitazoxanide (NTZ), initially identified as an antiparasitic drug which is used in humans, showed dose-dependent antiviral activity in vitro against different strains of FCV and a synergistic effect was observed with mizoribine (MZR), an imidazole nucleoside with antiproliferative activity against some immune cells [148]. Both substances had low cytotoxicity in vitro. Moreover, in experimentally FCV-infected cats, oral NTZ treatment three days post-infection (5–20 mg/kg suspended in 500 µL phosphate buffered saline) significantly reduced viral loads in the trachea and lungs, FCV RNA shedding and clinical signs of FCV infection as well as mortality [148]. No diarrhoea or vomiting was observed in treated cats despite these having been reported previously as potential side effects [148]. The authors concluded that NTZ could be used as therapeutic agents to treat FCV infection and that further studies should address potential synergy between NTZ and MZR in vivo, as well as the potential development of drug resistance.

A commercially available product, Feliserin (hyperimmune serum to the authors’ knowledge only available in Germany and in Austria available by special import) that contains antibodies against FCV, FHV, and feline parvovirus (FPV) is available commercially for the treatment of acute viral feline upper respiratory tract disease (FURTD) disease. One study evaluated its efficacy, compared to a placebo, for the treatment of acute viral FURTD due to FCV and/or FHV infection [88]. Feliserin was given for three days, subcutaneously once daily and topically into the eyes, nostrils, and mouth q8h. Clinical signs and health status in both groups improved significantly over time. Cats receiving hyperimmune serum significantly improved in terms of clinical signs by day 3, while cats in the placebo group only improved significantly by day 7. Thus, administration of hyperimmune serum lead to a more rapid improvement of clinical signs in cats with acute FURTD, but by day 7, clinical signs had improved equally in both groups, so the value of the treatment might not be sufficient to warrant its use.

### 8.3. Treatment of Cats with FCGS

A full description of the treatment of FCGS is beyond the scope of these guidelines. However, several modalities have been used to treat FCGS. Recommended options depend on disease severity and stage and include antibiotics plus rigorous dental cleaning, daily chlorhexidine application, corticosteroids and/or other immunosuppressant or immunomodulatory drugs (chlorambucil and cyclosporine [149]) and full teeth extractions [150]. Two studies investigated treatment of FCGS using intravenous injections of fresh adipose-derived mesenchymal stem cells; autologous cells were found to be more efficient than allogenic cells [151,152]. Five of seven cats completing the study responded to treatment by either complete clinical remission (*n* = 3) or substantial clinical improvement (*n* = 2) [152].

Anecdotal and clinical case reports and some studies have suggested the use of both feline interferon-ω and human interferons to treat cats with FCGS associated with FCV shedding, by intralesional or combined systemic plus intralesional application. A cat with FCGS, which had not responded completely to full teeth extraction, responded to treatment with feline interferon-ω within six weeks [153]. Feline interferon-ω was initially administered subcutaneously on alternate days at a dosage of 1 mega unit/kg for five doses, and then switched to 10^5^ IU/cat q24h PO (in 2 mL of isotonic saline solution) for two months and then q48h [153]. In a randomised, double-blinded study of 39 FCV-infected cats with FCGS, treatment with oral feline interferon-ω at 10^5^/cat q24h for 90 days was compared to treatment with prednisolone at 1 mg/kg q24h PO for seven days, followed by 1 mg/kg q48h PO for seven days, and finally 0.5 mg/kg q48h PO for seven days [154]. There was no significant difference between the two groups for most of the parameters, except for the pain score that was significantly lower in cats treated with feline interferon-ω after two and three months [154]. In another much shorter duration study, 17 FCV-infected cats with FCGS were treated with either feline interferon-ω SC at days 1, 2, 3, 7, 8, 14, and 21 (*n* = 13) or 1 mg/kg prednisolone q24h SC (*n* = 4) at the same time points [155]. There was a significant improvement in the clinical and salivation scores of the cats treated with feline interferon-ω, but neither in the pain upon opening the mouth, nor in the presence of halitosis or mandibular lymphadenopathy [155]. Oral administration of feline interferon-ω lead to clinical improvement of lesions in two cats with type II diabetes mellitus and concurrent FCGS, and the required insulin dose could also be decreased [156]. Thus, feline interferon-ω might be a good alternative to glucocorticoid treatment especially in cats in which full teeth extraction is not an option or in which glucocorticoid treatment is contraindicated, such as those with diabetes.

### 8.4. Other Management Considerations for FCV Infection

- Avoid stress, consider environmental enrichment and management in multicat households. One study looked at the effect of a synthetic feline facial pheromone in two shelters in the USA [157] in reducing stress scores and/or the incidence of infections associated with FURTD, compared to placebo, but no evidence was found that the pheromone product had any effect on stress scores or incidence of infections associated with FURTD in the shelter-housed cats.
- Consider hygiene, partitions, grouping, order of cleaning (deal with ill cats last), etc. Effective barrier nursing is essential for hospitalised patients being treated with acute infections associated with FURTD. However, staff should be mindful that outwardly normal cats could be shedding FCV.
- Be careful with introduction of new cats into a household, particularly if you have a FCV-free household; quarantine for three weeks with the option to swab for FCV before introduction into the household.
- If there are recurrent problems with FCV in a multicat environment, reduce the number of cats within the individual group (see also “Managing FCV outbreaks in multicat communities” [158]).

### 8.5. Treatment of Cats with VS-FCV Infection

In outbreaks of VS-FCV, severely affected cats have been treated with intensive care supportive treatment (e.g., fluid therapy and antibiotics) as well as with feline interferon-ω, and specific antibody preparations; with this, clinical improvement has been reported anecdotally in some cats. In some outbreaks, cats also received glucocorticoids; the usefulness of this treatment is controversial. Controlled clinical studies have not been published; so specific treatment for the disease is not currently known [65]. 

In vitro efficacy of novel antivirals was tested against VS-FCV from three Australian disease outbreaks and dose–response inhibition was obtained with several antivirals, including NTZ at low micromolar concentrations; further evaluation of the safety and efficacy of NTZ as a specific treatment option for FCV-VSD will be necessary [35].

## 9. General Recommendations on Vaccine Type and Vaccination Protocol

FCV infection is ubiquitous and can induce severe disease. The ABCD therefore recommends that all cats should be vaccinated against FCV [159]. European surveys of vaccine history in cats and owners’ attitudes to vaccination have shown that 69% of the cats in the UK had been vaccinated during the last 12 months in 2008 [160] and 78% of German cats had been vaccinated during the last three years in 2014/2015 [161]. Although vaccination provides good protection against FCV-associated acute oral and upper respiratory tract disease in most cases, it does not prevent cats from becoming infected or from shedding FCV following infection [162]. Nonetheless, some studies showed significantly lower infection rates in vaccinated compared to unvaccinated cats [2,86].

Currently, FCV is combined with only FHV in divalent vaccines (only available in some countries) or, more commonly, additionally with other antigens. Both modified live and inactivated parenteral vaccines are available. Modified live intranasal vaccines are still on the market in the USA, but unfortunately are no longer available in Europe. In one study, intranasal administration of an experimental inactivated, non-adjuvanted FCV vaccine has been shown to be better than a subcutaneous vaccine in terms of reducing clinical scores and virus shedding following exposure [29]. Moreover, six cats that had received an intranasal-intraocular vaccine in an experimental study did not develop detectable antibodies against feline renal cells, while six cats that were vaccinated parenterally demonstrated antibodies at multiple times during a 56 day follow-up period [163]. Thus, the authors suggested that subcutaneous vaccination (against FCV, FHV, and FPV) might have the potential to play a role in chronic kidney disease in cats, while intranasal vaccines are unlikely to have such a potential [163]. However, none of the cats developed notable renal inflammation, glomerular disease or urine abnormalities. Moreover, in 156 privately owned cats in Thailand, vaccinated animals did not have higher antibody levels against feline kidney extracts nor did they have antibodies binding to kidney tissue sections more frequently than unvaccinated cats, although the study did show a correlation between the presence of antibodies to vaccine antigens and feline kidney extracts [164]. Overall, there is insufficient evidence for a causal role of parenteral vaccination in the induction of renal disease and further studies are required.

The onset of immunity was determined to be within seven days after vaccination for a non-adjuvanted inactivated FCV vaccine: Kittens were protected from severe clinical signs and vaccine efficacy was similar seven days and four weeks after vaccination [165]. The duration of immunity was reported to be three years [166].

The impact of vaccination on the shedding of field viruses is controversial, with some studies showing a moderate reduction [83,165,167] whilst others show that vaccination might actually extend the period of virus shedding after infection [34,168,169]. One study reported that the impact of vaccination on shedding of FCV field strains depended on the time point after vaccination at which the cats were challenged; whilst a reduction in FCV shedding was observed when FCV infection occurred a few weeks or one year after vaccination, viral shedding was not reduced anymore with FCV infection three years after vaccination [166].

Live parenteral and intranasal FCV vaccine strains can be shed following vaccination, although this seems to be uncommon [9,45,50,116,169,170].

Live vaccines retain some pathogenic potential and can induce disease especially if administered incorrectly, e.g., when such a vaccine is accidentally aerosolised or spilled onto the cat’s skin or fur and ingested during grooming [50,169,170,171].

Cats that have recovered from FCV-associated disease are probably not protected for life against further episodes of disease, particularly those caused by different strains. Therefore, vaccination of recovered, healthy cats is generally recommended, even in situations where FCV is endemic, and vaccination should be applied as soon as the clinical signs have resolved to potentially induce immunity against a broader spectrum of strains.

A reduction in the volume for convenience of administration of multivalent vaccine preparations including FCV resulted in less immediate swelling reaction at the injection site, while retaining similar immunogenicity for FCV, in an experimental trial comparing ten cats that received the commercial vaccine (1 mL) and ten cats that received the novel 0.5 mL preparation [172]. Safety was also investigated in a field trial with 297 cats given the 0.5 mL and 101 cats given the 1 mL preparation; no significant difference was reported for immediate adverse reactions [172].

In contrast to FPV, the value of antibody testing in predicting protection is limited, because antibodies to the FCV strain detected in a laboratory test might not necessarily protect against the FCV strains to which the cat will subsequently be exposed in the field. Moreover, protection against FCV has been related also to cellular immunity [82,85,173]. Accordingly, a field study came to the conclusion that the value of FCV antibody testing in predicting protection from FCV infection and as a pre-vaccination test is limited and cannot replace routine vaccination against FCV [129].

### 9.1. Different FCV Strains

There is currently no vaccine available that protects equally well against all FCV field strains and vaccine companies are seeking to identify newer FCV strains that potentially provide wider cross protection. The most used vaccine strains are F9, which is the oldest and was isolated in the 1950s, FCV 255, and two newer strains G1 and 431 [26,83]. Some vaccine companies do not state the strain of virus used in their vaccine. One manufacturer has introduced a virulent strain into its vaccine in the USA [174]. While the vaccine protected from severe clinical signs due to challenge with the VS-FCV homologous to the vaccine strain, it is unknown how well this vaccine would protect cats from heterologous VS-FCV infection. A Japanese research group has developed a triple strain vaccine [175]; however, at the time of writing, the latter two vaccines are not available in Europe. As FCV can mutate quickly, field strains could theoretically evolve resistance to any vaccine-induced immune response, particularly if a vaccine is used for a prolonged period of time in the population [176].

Protection against FCV has been related to humoral (neutralising antibodies) and cellular immunity [82,85,173]. Therefore, it is difficult to make a general recommendation about which vaccine strain or strains to use based solely on results from in vitro neutralisation studies. Moreover, conflicting results have been published from studies testing the susceptibility of FCV field strains to neutralisation by sera raised by different vaccine strains. Three in vitro studies suggest that the oldest vaccine strains still effectively cross neutralise current field strains [73,177,178], whereas other studies demonstrated that antisera raised against newer vaccine strains effectively cross-neutralised a larger proportion of field strains compared to antisera raised against the oldest vaccine strain [179,180,181]. The different outcomes of these studies likely reflect both the different populations of cats that were sampled to isolate field strains, as well as the different methodologies adopted to prepare antisera against the vaccine strains.

Several studies were conducted to obtain more information about the field strains circulating in Europe and China [28,32,86,107,177,182]. A study in the UK addressed the hypothesis that the decades-long use of the FCV vaccine strain F9 drove the emergence of vaccine-resistant viruses [73,177]. The authors concluded that this was not the case as they observed no evidence of progressive phylogenetic or antigenic divergence from the vaccine strain F9 of geographically representative field isolates collected in 2001 and 2013/2014 [73,177]. Epidemiological and phylogenetic studies in Switzerland also indicated that FCV F9 clusters with contemporary FCV field isolates and provides protection against FCV infection under field conditions [2,32,44]. Moreover, in an in vivo study, modified live FCV F9 vaccination was shown to elicit a cellular cross-reactive immunity against a heterologous FCV field strain and to reduce viral RNA loads, duration of RNAemia, and the severity of clinical signs following challenge [82,183]. Likewise, in a comparative in vivo study, no evidence was found of reduced protection against a European FCV field strain of a FCV F9 strain-based vaccine in comparison with a FCV vaccine containing the newer G1 and 432 FCV strains [167]. A unique situation was present in China, where all commercial FCV vaccines were based on FCV 255, when a large study in 516 cats with clinical signs compatible with FCV infection demonstrated that properly vaccinated cats were approximately 2.9-fold less likely to be FCV infected compared to unvaccinated cats [86]. The authors nonetheless concluded that the FCV 255 vaccine efficacy was unsatisfactory.

Independent of the vaccine strains used, **if FCV-associated disease is found to be occurring in fully vaccinated cats then changing to a different FCV vaccine strain should be considered**.

### 9.2. Vaccination and VS-FC Infection

Vaccination did not protect cats against field infections in many VS-FCV outbreaks [3,57,105], although, experimentally, some protection has been shown and prior vaccination resulted in a milder form of the disease [8,84,181,184]. It is unknown whether this lack of, or insufficient, vaccine protection is due to inherent characteristics of highly virulent strains or whether vaccine-“susceptible” strains are unlikely to cause outbreaks since vaccination is so widely practiced [8,65]. However, at least in one study it was recognised that many of the affected cats had not been, or had been insufficiently, vaccinated against FCV and that, according to in vitro neutralisation patterns, some cases might have potentially been prevented by vaccination [102]. Moreover, in a large VS-FCV outbreak at the University Animal Hospital of Zurich, Switzerland, in 2016, most of the affected cats (approx. 80%) had not been FCV vaccinated or had been vaccinated insufficiently (R.H.-L. personal communication).

### 9.3. Primary Vaccination Course

- **Kittens:** The ABCD recommends that all kittens should be vaccinated against FCV [159] (Table 1). MDA can interfere with the response to vaccination, and thus, the primary course of vaccination is usually started at approximately nine weeks of age, although some vaccines are licensed for use at an earlier age. Kittens should receive a second vaccination two to four weeks later, but not earlier than at 12 weeks of age. This protocol has been developed to ensure optimal protection. However, due to a longer persistence of MDA, some kittens might fail to respond to this protocol [77,185]. Therefore, in high-risk situations, particularly where FCV has been shown to cause disease in vaccinated kittens as well as if presence of high MDA is expected in the kittens, a third vaccination at 16 weeks should be considered. After the kitten primary vaccination course, all cats should receive an additional vaccine dose at 10 to 16 months of age: This will ensure adequate vaccine-induced immunity for cats that might not have adequately responded to the primary vaccination course. It is recommended that the same vaccine brand or at least the same vaccine strain(s) is used for the entire primary vaccination course.
- **Older cats** of uncertain FCV vaccination status should also receive two injections with an interval of two to four weeks, and a boost one year later, using vaccines containing the same virus strains (Table 1). This applies even if the vaccine contains modified live virus.

### 9.4. Revaccinations

The issue of recommended intervals between boosters is still controversial. However, based on study results published by several independent groups, the ABCD recommends that boosters should be given at triennial intervals to protect individual cats against FCV field infections for cats in low-risk situations, such as mainly indoor-only cats with little or no contact with other cats (Table 1). However, owners should be made aware that as time since the last vaccination increases, the degree of protection from development of clinical signs decreases.

However, cats in crowded high-risk situations (e.g., boarding catteries) should be revaccinated at yearly intervals (Table 1). For other cats, an informed decision should be made based on a risk–benefit analysis. The ABCD recommends a single injection if the interval since the last vaccination is not more than three years. Even if the interval exceeds three years, usually one vaccination would ensure protection, especially if a modified live vaccine is used. When switching FCV vaccines to different vaccine strains for boosters, then one vaccine is sufficient if the new vaccine contains a modified life strain; however, two vaccines are recommended to ensure optimal protection if switching to an inactivated vaccine, when the inactivated vaccine contains different strains to the previous vaccine. The ABCD realises that unfortunately single-component FCV vaccines are currently unavailable. Annual boosters that protect against other antigens might in practice entail more frequent boosters than triennially.

## 10. Disease Control in Specific Situations

In multicat environments, FCV frequently causes problems, and disease control may focus on how much FCV-associated disease is tolerable in that situation. To reduce FCV-associated clinical problems in multicat environments, special measures are necessary, such as decreasing cat group sizes, reducing introductions of new cats and quarantining newly introduced cats, separating sick cats, hygienic measures using disinfection effective against FCV and washing clothes at temperatures ≥ 60 °C, and augmenting vaccination to annual boosters and changing vaccine strain if disease persists [159]. The ABCD has developed a factsheet aimed at veterinarians in practice responsible for the health and wellbeing of groups of cats that helps develop structured healthcare discussions with clients and locate weak points that can be improved to reduce FCV problems within the facility [158].

### 10.1. Shelters

FCV is often a problem in cat shelters. Management to limit or even prevent virus transmission is as important as vaccination in control. Shelter design and management should be aimed at avoiding cross infection of cats. Cats should be housed individually unless they are known to originate from the same household. Dogs and cats should be housed separately, and flea control should be implemented to minimise the risk of transmission of FCV and other infections. If acute respiratory disease occurs in a shelter, identification of the agent involved (with differentiation of FCV from FHV, *Chlamydia felis*, *Bordetella bronchiseptica*, and *Mycoplasma* spp.) is useful in deciding on the appropriate preventative measures and treatment. In case of an FCV outbreak, it should be considered that FCV can persist in the environment for about one month [54,55,56] and is resistant to many common disinfectants. Effective substances include sodium hypochlorite (2700 ppm; 1 min), potassium peroxy-monosulphate (1%; 10 min), chlorine dioxide (10 ppm) [186,187], and commercial products that have been approved for their virucidal activity against noroviruses. Any clothing or cat bedding, etc., should be washed at ≥60 °C. New healthy cats should be vaccinated as soon as possible [159]. Modified live virus vaccines are preferred in shelters because of their earlier onset of protection.

### 10.2. Breeding Catteries

FCV can be a major problem for cat breeders. Infection most often appears as FURTD in young kittens, typically at approximately four to eight weeks as MDA wane. Disease in such young kittens can be severe and frequently involves all the kittens in the litter; some kittens can die. Vaccination of the queen will not prevent virus shedding but can be beneficial in ensuring that the kittens benefit from higher levels of MDA through the colostrum and milk, providing protection for the first month or so of life.

Revaccinations of queens should take place prior to mating. Vaccination during pregnancy is not recommended. Modified live virus vaccines are not licensed for use in pregnant cats and if considered at all, an inactivated vaccine should be used. Queens should kitten in isolation, and to avoid the risk of exposure to potential carrier cats, the litter should not mix with other cats until it has been fully vaccinated. Early vaccination should be considered for litters from queens that had infected litters previously or for which there is concern of infection [159]. The earliest age for which FCV vaccines are licensed is six weeks, but vaccination could be considered even earlier in kittens deemed to be at risk. When levels of MDA might be too low to protect, vaccination should be repeated every two weeks until the primary vaccination course is concluded at 12 weeks; a further vaccine at 16 weeks is not required if levels of MDA are known to be low.

When all other control strategies have failed, early weaning into isolation from approximately four weeks of age has been suggested, as an alternative approach to protect kittens against infection from their mothers. However, the stress and behavioural problems that arise from early weaning can be huge. Therefore, and since there is no evidence to support its efficacy, the ABCD does not recommend early weaning for the prevention of FCV infection and disease.

### 10.3. VS-FCV Outbreaks

If VS-FCV outbreaks are encountered, this usually happens in multicat environments, such as cat shelters or veterinary facilities. Controlling a VS-FCV outbreak is very challenging; all exposed cats must be considered at risk for transmitting severe disease to others; there is no clearly defined “quarantine period” after which it is known that cats are safe; and it cannot be assumed that vaccination is protective. Moreover, indirect transmission must be considered through persons and fomites [8,57,58,59]. It is important to create an immediate and complete temporal break between exposed cats and newly admitted cats to the shelter or veterinary facility. To achieve this, veterinary practices/clinics have been completely closed for cats following an outbreak for at least one month; this was effective in controlling the outbreaks in the past. The exposed cats should be isolated/quarantined. Hygiene and disinfection are very important, including thoroughly cleaning of all surfaces, followed by application of a disinfectant active against non-enveloped viruses, e.g., accelerated hydrogen peroxide (35,000 ppm, 10 min), potassium peroxy-monosulphate (1%, 10 min), and sodium hypochlorite (2700 ppm; 1 min) or aldehydes (2%) are all effective [186,187,188]. Pre-cleaning with a detergent to remove all organic matter is important when using chemical disinfectants. For more information on disinfectant choice in feline veterinary hospitals, shelters and cat households and managing outbreaks of VS-FCV infection in shelters, helpful online information resources are available [188,189].

## 11. Conclusions

FCV is highly prevalent and poses problems mainly in multicat environments and when hygienic conditions are suboptimal. Elimination of this infection from multicat groups is very difficult. The high tenacity of FCV necessitates strict hygienic measures and the use of disinfectants that efficaciously eliminate FCV in the environment based on sodium hypochlorite, potassium peroxy-monosulphate and chlorine dioxide. Keeping cats in stable isolated groups of not more than three animals can help reduce problems resulting from infection by FCV (and other agents as well). Vaccination of cats in multicat environments should adopt yearly schedules. The high antigenic variability of FCV poses a challenge for vaccine development and several attempts have been made to produce broadly neutralising vaccines. Efficacious vaccines usually prevent FCV-associated disease but not infection, and vaccine efficacy varies on the prevailing FCV field strain. Therefore, if problems persist in fully vaccinated cats, a change of the vaccine strain(s) should be considered. Moreover, FCV vaccines have lower efficacy against highly virulent strains. Ideally, further efforts will aim to develop FCV vaccines that prevent infection, or at least all potential detrimental clinical manifestations.

This guideline will continue to be updated regularly on the ABCD homepage (www.abcdcatsvets.org) as new data become available. A previous version of this ABCD guideline was published in 2009 [190]. The current review is an update of this previous version.

## Figures and Tables

**Table 1:** Recommended FCV vaccination course in outdoor cats and indoor-only cats, rescue shelters cats and breeding cats (FCV is usually vaccinated in combination with FPV and FHV) (according to [159]). These vaccine recommendations are updated regularly and the latest version can be downloaded at [159].

	Primary Vaccination 1	Primary Vaccination 2	Primary Vaccination 3	Final Primary Vaccination	Boosters
**Outdoor cats and indoor-only cats** ^1^					
Kittens (age)	8 to 9 weeks	12 weeks	16 weeks	10 to 16 months	Annually (or up to every 3 years in low-risk situations)
Adult cat vaccinated <3 years ago	One immunisation	-	-	-	Annually (or up to every 3 years in low-risk situations)
Adult cat vaccinated ≥3 years ago	First immunisation	Second immunisation 2 to 4 weeks later	-	-	Annually (or up to every 3 years in low-risk situations)
Adult cat with no/unknown vaccination history	First immunisation	Second immunisation 2 to 4 weeks later	-	-	Annually (or up to every 3 years in low-risk situations)
**Rescue shelter cats** ^1,2^					
Kittens (age)	6 weeks	3 to 4 weeks later	3 to 4 weeks later until 16 weeks	10 to 16 months	Annually
Adult cat vaccinated <3 years ago	One immunisation	-	-	-	Annually
Adult cat vaccinated ≥3 years ago	First immunisation	Second immunisation 2 to 4 weeks later	-	-	Annually
Adult cat with no/unknown vaccination history	First immunisation	Second immunisation 2 to 4 weeks later	-	-	Annually
**Breeding cats** ^1^					
Kittens (age) ^3^	8 to 9 weeks	12 weeks	16 weeks	10 to 16 months	Annually
Queens	-	-	-	-	Annually and before breeding if low MDA

^1^ Recovered cats should still be vaccinated. ^2^ Vaccinate new incoming cats as soon as possible. ^3^ Consider earlier vaccination in litters from queens that have had infected litters previously. In high-risk situations, kittens can be vaccinated from 4 to 6 weeks of age, then every 2 weeks until 12 or 16 weeks of age. MDA = maternally derived antibodies.
